# Defined media reveal the essential role of lipid scavenging in supporting cancer cell proliferation

**DOI:** 10.1016/j.jbc.2025.110693

**Published:** 2025-09-08

**Authors:** Oliver J. Newsom, Eric Zheng, Lucas B. Sullivan

**Affiliations:** Human Biology Division, Fred Hutchinson Cancer Center, Seattle, Washington, USA

**Keywords:** cancer metabolism, cell culture, metabolomics, cancer biology, lipid metabolism, serum, lipid scavenging

## Abstract

Fetal bovine serum (FBS) is an undefined additive that is ubiquitous to mammalian cell culture media and whose functional contributions to promoting cell proliferation remain poorly understood. Efforts to replace serum supplementation in culture media have been hindered by an incomplete understanding of the environmental requirements fulfilled by FBS. Here, we use a combination of live-cell imaging and quantitative lipidomics to elucidate the role of serum in supporting proliferation. We show that serum provides consumed factors that enable proliferation, with serum metal and lipid components serving as crucial metabolic resources. Despite access to a wide range of lipid classes available in serum, we find albumin-bound lipids are the primary species consumed by cancer cells. Furthermore, we find that supplementing with additives that contain necessary metals and any of the albumin-associated lipid classes can obviate the FBS requirement for cancer cell proliferation. Using this defined system, we investigated cancer cell lipid consumption dynamics, finding that albumin-associated lipids are primarily consumed through a mass-action mechanism with minimal competition within or amongst lipid classes. We also find that lipid scavenging is a dominant lipid acquisition route and is necessary for cancer cell proliferation. This work therefore identifies metabolic contributions of serum and provides a framework for building defined culture systems that sustain cell proliferation without the undefined contributions of serum.

Since the origins of cell culture, animal serum has been an essential additive for supporting robust and sustained growth of most cell lines (1, 2). Among the various serum additives used in culture media, fetal bovine serum (FBS) is the most widely used in biomedical research, primarily attributed to its roles in providing a complex mix of growth factors, lipids, and other nutrients (2, 3). However, despite the ubiquitous use of FBS, its fundamentally undefined nature poses a challenge for scientific understanding, since the many biologically active components cannot be readily studied in isolation using traditional culture media formulations. In addition, the inherent batch-to-batch variability of FBS can influence experimental outcomes and thus contribute to reproducibility issues (4, 5). Aside from its biological influence, FBS also has the potential to introduce zoonotic pathogens into cell culture systems, resulting in safety concerns, particularly in the context of biomanufacturing (6). Finally, it is unclear whether fetal bovine fluid is relevant for studying postnatal human disease, as its composition may inappropriately influence biological phenotypes and inadequately model the *in vivo* environment. These issues therefore highlight the need for robust serum-free alternatives for cell culture.

A major hurdle to excluding FBS from cell culture media is that the serum components required to sustain cell proliferation remain poorly defined. For instance, although growth factors are a well-known constituent of FBS, the extent to which specific serum-derived hormones drive cell proliferation remains uncertain (7). In the case of cancer cell culture, the requirement for fetal serum rich in growth factors is questionable, as a hallmark of cancer is constitutive activation of growth factor signaling pathways in the absence of exogenous cues (8). Moreover, fetal growth factors may impair our understanding of oncogenic signaling in cancer by masking phenotypic effects of cell-intrinsic alterations to cancer-associated growth factor pathways. Alternatively, FBS may support cancer cell proliferation by providing small-molecule nutrients, including lipids, soluble metabolites, vitamins, and trace metals. Indeed, several studies have found that modifying the relative proportion of these molecules in media through various filtering, extraction, and formulation strategies can influence genetic and metabolic dependencies of proliferating cancer cells (9, 10, 11, 12). The potential nutritional contributions of FBS are also implied when considering that some biologically important vitamins (*e.g*., biotin and cobalamin) and metals (*e.g*., iron, zinc, and copper) are absent in some commonly used cell culture media formulations (10, 13). Indeed, culture media additives that support proliferation in serum-restricted conditions often include trace metals or the metal carrier protein transferrin (2, 14). Nonetheless, the development of a serum-free media that enables robust proliferation across a broad range of cell lines remains a challenge since the role of serum remains unclear. Together, these challenges highlight the need for culture media capable of supporting cell proliferation without the addition of serum, which would enable investigations into the metabolic and signaling factors that influence proliferation that are otherwise masked by bioactive serum factors (5, 9). However, the essential components of serum required for proliferation remain incompletely defined. We hypothesize that serum provides key consumable factors that can be systematically replaced to create a defined serum-free media capable of supporting proliferation. Identifying serum components that fulfill proliferative requirements would advance the development of serum-free culture systems and broaden the range of cell lines that can be maintained without the reliance on serum-containing media formulations. Collectively, these issues underscore the need for a minimal media capable of supporting cell proliferation without the addition of serum, which would facilitate investigations into the environmental requirements for proliferation, including the metabolic and signaling factors that are otherwise inextricably fulfilled by the numerous serum components.

To explore environmental dependencies of serum for cell proliferation in culture, we combined live cell imaging with LC–MS to investigate the role of FBS components—particularly serum lipids—in supporting cancer cell proliferation. We developed a defined serum-free media to explore and compare the metabolic contributions of serum, particularly serum lipids, during cancer cell proliferation. Despite the broad range of lipid species in FBS, our findings reveal that cells selectively consume albumin-associated lipid classes during proliferation, whereas lipids transported in lipoprotein complexes were minimally depleted. Furthermore, FBS could be replaced from culture media with a combination of albumin-associated lipid classes and a defined additive mix containing absent metals, enabling the uncoupling of metabolic variables that are normally provided by FBS. Our results underscore the critical role of lipids in supporting cell growth and provide a platform to investigate lipid metabolism and other contributions of serum in the absence of confounding variables derived from undefined serum components.

## Results

### Serum provides essential consumable factors to support cell proliferation

FBS is a key additive to culture media for supporting cell proliferation, yet the mechanism by which serum restriction impairs cell proliferation remains incompletely understood. Cell proliferation measurements typically utilize initial and end-point cell counts to calculate proliferation rates while assuming a constant doubling time throughout the experiment, which is unlikely to be a reasonable assumption in all settings. We propose two hypothetical models of proliferation defects that can result in the same final cell count: (1) a rate-limitation phenotype, where restriction of growth-promoting factors results in a slowed but constant proliferation rate or (2) a depletion phenotype, where the initial proliferation rate is stable but decreases once essential factors are exhausted (Fig. S1*A*). Given that these two models reflect different biological mechanisms, we hypothesized that understanding the kinetics of proliferation defects during FBS restriction may inform how FBS supports cell proliferation.

**Figure 1:**
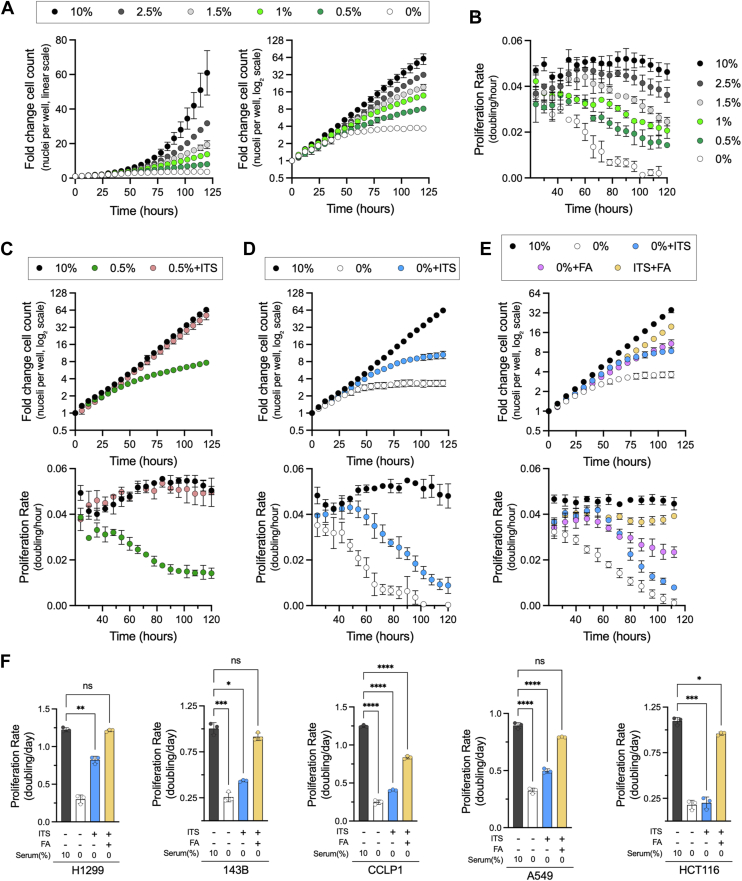
**Effects of serum limitation on cancer cell proliferation rates.***A*, fold change in H1299 NucRFP cell counts over time relative to time 0 (hours = 0), quantified by changes in nuclei count in media containing varying concentrations of FBS. Data are presented on both a linear scale (*left*) and a log_2_ scale (*right*). *B*, moving average of the proliferation rate, calculated between consecutive time points and averaged over 24-h intervals, corresponding to cell nuclei counts from (*A*). *C*, fold change in cell counts (*top*) and moving average of proliferation rate (*bottom*) in H1299 NucRFP cells cultured in 10% or 0.5% FBS and treated with either vehicle or ITS. *D*, fold change in cell counts (*top*) and moving average of proliferation rate *(bottom*) in H1299 NucRFP cells cultured in 10% FBS or DMEM alone, with or without ITS treatment. *E*, fold change in cell counts (*top*) and moving average of proliferation rate (*bottom*) in H1299 NucRFP cells cultured in 10% FBS or DMEM alone, treated with FA mix (100 μM), ITS, or both. *F*, average proliferation rates of H1299, 143B, CCLP1, A549, and HCT116 cells cultured in 10% FBS, ITS alone, or ITS combined with FA mix, calculated using initial and final cell counts. Error bars indicate mean ± SD (n = 3). Statistical significance was assessed using Brown–Forsythe and Welch ANOVA tests (*F*). ns = not significant, ∗*p* < 0.05, and ∗∗∗∗*p* < 0.0001. DMEM, Dulbecco's modified Eagle's medium; FA, fatty acid; FBS, fetal bovine serum; ITS, insulin–transferrin–selenium.

To investigate the contribution of FBS to proliferation, we expressed nuclear-localized red fluorescent protein in H1299 non–small cell lung cancer cells (H1299 NucRFP) and used live-cell imaging to track nuclei counts in real time as a direct readout of cell proliferation in Dulbecco's modified Eagle's medium (DMEM) with various FBS concentrations. As expected, FBS was required for sustained cell proliferation, and final cell counts were proportional to FBS media concentration (Fig. 1*A*, *left*). Interestingly, when comparing the kinetics of cell population growth over time, all FBS concentrations shared similar increases in cell counts at early time points, but the rate of cell population growth slowed over time proportional to FBS concentrations (Fig. 1*A*, *right*). When we converted changes in cell count to time-resolved proliferation rates, the kinetics closely matched those predicted for a depletion phenotype (Figs. 1*B* and S1*A*). Notably, the rate of proliferation decay was comparable in each case of FBS limitation, with the primary difference being that the onset of proliferation defects occurred later in conditions with higher FBS concentrations (Fig. 1*B*). These findings therefore support the hypothesis that FBS restriction impairs cell proliferation by limiting consumable components necessary to sustain proliferation.

Since basal media lack numerous components found in FBS that could support proliferation, we next tested whether supplementation with a defined additive containing insulin, transferrin, selenium, ethanolamine, and biologically relevant metals (insulin–transferrin–selenium [ITS], see *Experimental procedures* section), a mixture shown to support cell proliferation in serum-restricted conditions, could similarly restore cell proliferation to these cells during FBS limitation (15, 16). Indeed, ITS was sufficient to fully restore the proliferation rate kinetics of H1299 NucRFP cells cultured in serum-restricted conditions (0.5% FBS), matching that of cells cultured in 10% FBS (Fig. 1*C*). We next investigated which component(s) of the ITS mixture mediated the proliferation restoration in low FBS conditions and observed that transferrin and metals are the dominant contributors to H1299 NucRFP proliferation upon serum restriction (Fig. S1*B*). Consistent with the limitation of these micronutrients in low serum conditions, titrating down the concentration of ITS in 0.5% FBS restored the depletion phenotype (Fig. S1*C*). These data therefore indicate that a primary limitation in serum-restricted conditions is the depletion of trace metals that are not otherwise provided in the basal media. We next tested whether adding ITS could replace FBS altogether. Although ITS improved the overall number of doublings compared with DMEM alone, these cells still exhibited a decaying proliferation rate indicative of a delayed depletion phenotype (Fig. 1*D*). This suggests that while ITS is beneficial under reduced FBS conditions, presumably by solving the first-order deficiency of metals required for growth, it cannot replace FBS entirely, as it lacks additional essential serum components that are required to sustain cell proliferation.

We next explored how other factors in FBS may contribute to cell proliferation of ITS-treated cells in basal media. Serum-derived lipids serve as a metabolic resource that is utilized during cell proliferation and can be conditionally essential during metabolic stress (11, 17, 18, 19). Thus, we tested the impact of lipid supplementation by adding a fatty acid (FA) mix composed of palmitate and oleate, the two most abundant saturated and monounsaturated FA species in serum, conjugated to bovine serum albumin (BSA) (20, 21). Like ITS, the FA mix improved overall cell proliferation relative to basal media alone, but the cell proliferation rate eventually decayed over time (Fig. 1*E*). Importantly, the cosupplementation of basal media with FA mix and ITS, or just the metals/transferrin constituents of ITS, was sufficient to prevent the cell proliferation rate decay and maintain exponential growth in the absence of FBS, albeit with a modest rate limitation phenotype compared with cells cultured in 10% FBS (Figs. 1*E* and S1*D*). We also investigated if this combination could support the proliferation of a diverse group of cancer cell lines, including cells deriving from non–small cell lung cancer (A549), osteosarcoma (143B), cholangiocarcinoma (CCLP1), and colon cancer (HCT116). Measuring proliferation rates by cell count, we observed that the combination of ITS and FA mix was sufficient to support rapid serum-free cell proliferation in each cell line (Fig. 1*F*). The addition of FBS to culture media still adds convenience for various cell culture functions, including inactivating trypsin while splitting adherent cells and providing attachment factors that encourage cells to adhere to culture dishes, making long-term culture of adherent cells in serum-free culture difficult to evaluate. Nonetheless, we found that this FBS replacement formulation can support robust cell proliferation of Jurkat suspension cells for greater than 4 weeks (Fig. S2, *A* and *B*). These findings therefore highlight that the requirement for serum can be largely obviated for cancer cell culture experiments by providing a source of metals and FAs, which are otherwise absent from basal media.

Growth factors are thought to be key serum constituents that can contribute to driving cell proliferation. The finding that cancer cells can proliferate in serum-free media without exogenous growth factors thus aligns with the concept that transformed cells have intrinsic genetic changes that maintain growth factor signaling without external cues (8). However, serum-containing media are also used to support the proliferation of nontransformed cells, raising the possibility that these noncancerous cells may still be reliant on the growth factor components of FBS. Thus, we assessed whether our defined media were sufficient to support proliferation of nontransformed cells, including human foreskin fibroblast (HFF-1), immortalized mouse myoblast cells (C2C12), and human airway epithelial cells (AALE). While both ITS and FA mix were beneficial to these nontransformed cells in serum-free conditions, the combination failed to support cell proliferation as robustly as in transformed cells (Figs. 1*F* and S2*C*). Indeed, when we calculated the relative proliferation rate of each cell line grown in the ITS–FA mix containing serum-free media relative to standard FBS-containing conditions, the proliferation rates of transformed lines were better maintained in serum-free media conditions compared with nontransformed lines (Fig. S2*D*). These results indicate that our defined media satisfy the metabolic dependencies of cell proliferation but lack critical components, likely growth factors, that are required for the proliferation of nontransformed cell lines.

### FBS lipidome and its consumption by proliferating cells

**Figure 2:**
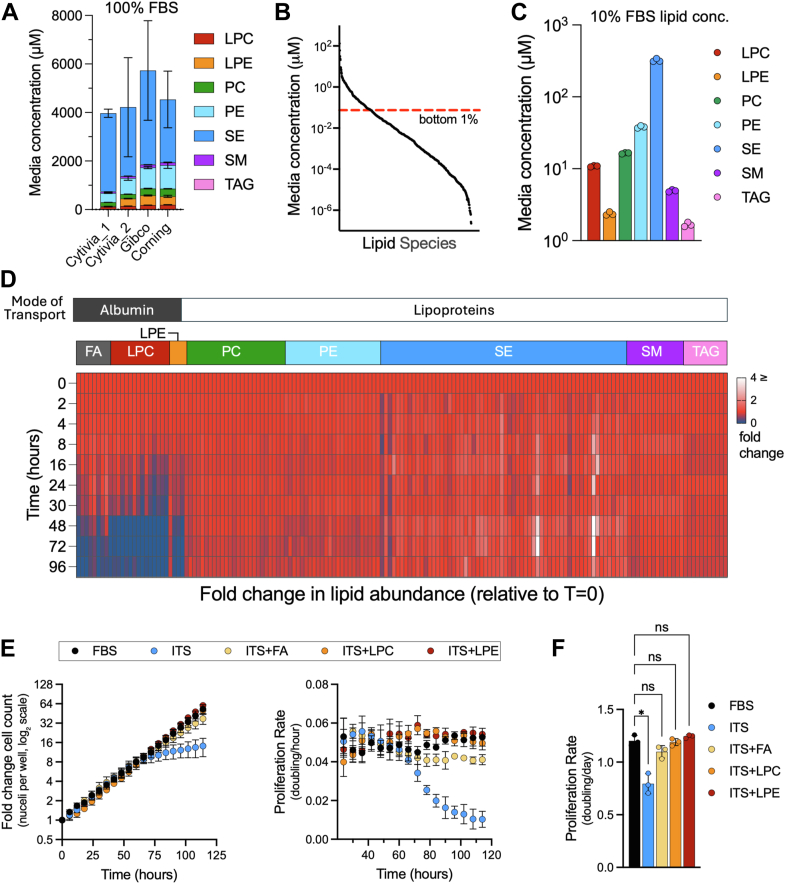
**Albumin-associated lipids are consumed during cancer cell proliferation.***A*, comparison of lipid profiles across multiple lots of commercially available FBS (100% serum). *B*, lipid species detected in media with 10% FBS (Cytiva Lot #1), ranked by abundance. Lipids below the *red line* represent the bottom 1% of total lipid content. *C*, concentrations of individual lipid classes in media with 10% FBS used for subsequent experiments. *D*, fold change in concentration of the most abundant lipids, normalized to initial concentrations in media containing 10% FBS during cell proliferation. Each column represents individual lipid species over time, and the color indicates relative fold change: depleted (*blue*), no change (*red*), and increased (*white*). *E*, fold change in cell counts (*left*) and moving average of proliferation rate (*right*) in H1299 cells cultured in either 10% FBS or in serum-free DMEM supplemented with ITS and either LPC, LPE, or FA mix. *F*, average proliferation rate of cells over the entire assay. Error bars represent mean ± SD (n = 3). Statistical significance was assessed using Brown–Forsythe and Welch ANOVA tests (*F*). ns = not significant, ∗*p* < 0.05. DMEM, Dulbecco's modified Eagle's medium; FA, fatty acid; FBS, fetal bovine serum; ITS, insulin–transferrin–selenium; LPC, lysophosphatidylcholine; LPE, lysophosphatidylethanolamine; PC, phosphatidylcholine; PE, phosphatidylethanolamine; SE, sterol ester; SM, sphingolipid; TAG, triacylglycerol.

While our data demonstrate that exogenous FAs can fulfill a major role of FBS in providing lipids to support growth in culture, FBS is a heterogeneous mixture containing a wide repertoire of lipid species, and so it remains unclear which serum lipids are consumed by cultured cells. Because multiple lipid species may redundantly support proliferation, we next examined the lipid composition of FBS and measured which lipids are consumed by proliferating cells. The serum lipidome includes diverse lipid species that vary in the number of FA chains, lipid head groups, as well as the length and saturation of the hydrocarbon chains (22). To characterize the major lipid classes in FBS and their variability across different FBS lots, we used semitargeted quantitative LC–MS to profile lipid metabolites in four different FBS lots (Fig. 2*A*). Consistent with prior reports, the most abundant lipid classes included neutral lipids, where sterol esters (SE) were much more abundant than triacylglycerols (TAGs) (23). In addition, polar lipids such as sphingomyelin (SM) and glycerophospholipids, including phosphatidylethanolamine (PE), phosphatidylcholine (PC), and their precursor lysolipids, lyso-PC (LPC) and lyso-PE (LPE), were also detected in all FBS samples. While each lipid class was consistently detected across FBS lots, the relative and total abundance of lipid classes varied between different lots of FBS. These findings therefore demonstrate the inherent variability of using serum in cell culture settings and highlight the need to explore the roles of FBS-derived lipids in a more controlled system.

We next sought to determine which serum lipids are consumed by proliferating H1299 NucRFP cells. To focus on the lipids present in FBS at biologically relevant concentrations for proliferation, we ranked the detected lipid species from the FBS lot used for the remaining experiments (Cytivia_1) from most abundant to least and excluded those that constituted the bottom 1% of the total available FBS lipid pool, narrowing the analysis from 646 species to 163 lipid species spread across eight classes, varying in FA chain length and degree of saturation (Figs. 2, *B*, *C* and S3). Among these, SEs were the most abundant lipid class, quantified at 3.22 ± 0.17 mM, whereas TAGs were the least abundant lipid class, at 16.73 ± 1.15 μM. PEs and PCs, the most abundant glycerophospholipids detected, were present at 381.93 ± 14.28 μM and 164.78 ± 3.74 μM, respectively, whereas SMs were detected at 51.25 ± 1.70 μM. The lipid precursors, LPC and LPE, were detected at lower concentrations of 108.69 ± 2.37 μM and 23.66 ± 1.28 μM, respectively. Free FAs, particularly saturated and monounsaturated species, are well-known background contaminants in lipidomics workflows that are introduced from solvents, plasticware, and sample handling (24, 25), and so were excluded from this serum lipid analysis.

To identify which FBS-derived lipids are consumed from the media during proliferation, we collected spent media from cells cultured in 10% FBS-containing media at multiple time points and profiled the change in the media lipidome over time. Due to high background contamination of oleate and palmitate, we added tracer amounts of stable isotope–labeled versions of each to monitor the depletion of their associated lipid pools without substantially altering the total FA pool. Other FAs with relatively little background interference were tracked directly. Notably, only a subset of all available lipids showed meaningful depletion relative to their starting concentration. Specifically, the lipid precursors containing only a single FA, which include FA, LPC, and LPE, were selectively consumed, with >80% depletion in most species relative to the starting amount over 96 h (Fig. 2*D*). In contrast, the complex lipids, including SE, TAG, PC, PE, and SM, exhibited minimal change with <5% depletion in most species during the same period. Notably, the pattern of media lipid consumption was closely associated with the mode of transport in serum. Indeed, highly consumed lipid species, including FA, LPC, and LPE, are primarily transported bound to BSA, whereas SE, TAG, PC, PE, and SM are carried in lipoprotein complexes (23, 26, 27, 28, 29). To determine whether selective albumin-associated lipid consumption is specific to H1299 NucRFP cells or represents a broader phenotype of proliferating cancer cells, we profiled media lipid consumption during growth across multiple cell lines. Notably, depletion patterns were broadly similar across all cell lines tested, with cells exhibiting selective depletion of albumin-bound lipids and minimal consumption of lipoprotein-associated lipids during proliferation (Fig. S4). Altogether, the preference for albumin-bound lipids during proliferation across various cells suggests that these lipids fulfill a general metabolic requirement for cancer cell proliferation in culture.

### Consumed lipid species redundantly fulfill the lipid requirements of serum

Our data indicate that FAs can fulfill an essential function of FBS in supporting cell proliferation, yet in the context of FBS-containing media, FAs are consumed alongside LPC and LPE lipid species. We thus sought to determine whether these albumin-associated lipid classes were redundant or distinct in their ability to support cell proliferation. We therefore cultured H1299 NucRFP in serum-free media containing ITS, with or without cosupplementation of individual albumin-associated lipid classes, and tracked their effect on proliferation kinetics (Fig. 2*E*). Notably, like the combination of ITS and FA mix, both the LPC and LPE lipid mixes were equally sufficient to fulfill the lipid demand for cell proliferation and prevent the depletion phenotype of cells cultured in the absence of serum. Indeed, the average proliferation rate was not statistically different between the cells grown in 10% FBS and any of these serum-free media (Fig. 2*F*). These data therefore indicate that albumin-associated lipid classes can redundantly fulfill the lipids needed to support proliferation.

### Lipid consumption dynamics are a function of environmental availability

**Figure 3:**
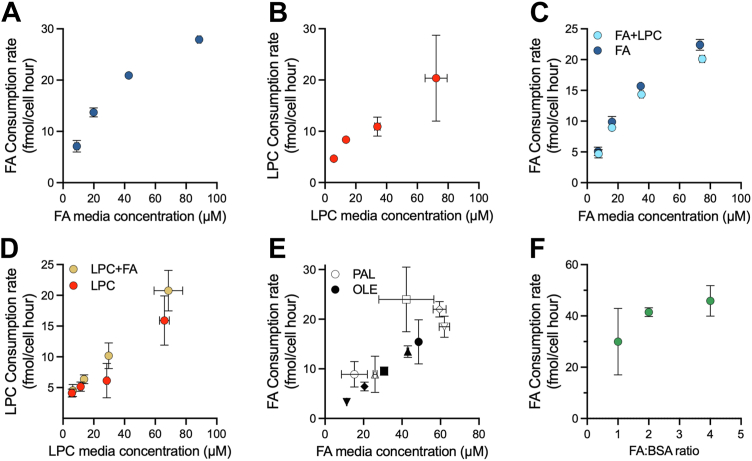
**Exploration of lipid scavenging kinetics in serum-free systems.***A* and *B*, FA and LPC consumption rates in cells cultured in serum-free media with varying FA (*A*) or LPC (*B*) concentrations. *C* and *D*, FA (*C*) and LPC (*D*) consumption rates from cells cultured in serum-free media containing an equimolar mixture of FA and LPC, compared with cells cultured in media containing only FA (*C*) or only LPC (*D*). *E*, palmitate and oleate consumption rates in cells cultured with a fixed total FA concentration and varying molar ratios of oleate:palmitate (indicated by matching shapes: ○ 4:1 △ 2:1 □ 1:1 ◇ 1:2 ▽ 1:4). *F*, FA consumption rates in cells cultured with a fixed FA concentration and varying concentrations of BSA; *shaded line* shows the 95% confidence interval of the regression line. Error bars represent mean ± SD (n = 3). BSA, bovine serum albumin; FA, fatty acid; LPC, lysophosphatidylcholine; OLE, oleate; PAL, palmitate.

The mechanisms and variables that dictate lipid consumption remain poorly understood. Thus, we used our serum-free media formulation as a tool to further explore the factors that influence lipid consumption of proliferating cells, using serum-free media containing FAs and/or LPCs as a lipid source. We first cultured cells across a dilution series of FA or LPC mixes and quantified consumption rates. Importantly, without FBS, our formulation enables us to use isotopically labeled FAs as the sole lipid source in the media, supporting more accurate quantification of lipid consumption by accounting for all exogenous lipids and avoiding interference from background FA contaminants. Similar to 10% FBS, cells grown in serum-free media readily consume exogenous FAs and LPCs (Fig. 3, *A* and *B*). Notably, the consumption rate for both FA and LPC increased proportionally with media concentration, indicating that lipid consumption is predominantly a function of concentration and that cells can scavenge lipids at increased rates when they are more abundant in the environment.

Our data indicate that lipid consumption rates within a lipid class are primarily concentration dependent, but it is unclear if consumption of one class influences consumption of other classes. We next sought to explore how the availability of FA-containing molecules, specifically FAs and LPCs, impacted the consumption of each other. We cultured cells across a dilution series containing an equimolar concentration of FAs and LPCs and compared the consumption rates to those measured when cells were grown in media containing a single lipid class (Fig. 3, *C* and *D*). Surprisingly, despite sharing BSA as a carrier molecule, FA and LPC uptake was unaffected by the presence of the other lipid class, further supporting noncompetitive, concentration-dependent lipid consumption dynamics for both lipid classes. To further explore lipid intrinsic variables affecting lipid consumption, we tested if consumption was selective for FAs based upon chain structure. We thus cultured H1299 nucRFP cells in a fixed concentration of total FAs while varying the molar ratio of palmitate to oleate and quantified consumption of each lipid individually. Interestingly, consumption of each FA followed a similar trend as other uptake variables—showing that, independent of FA chain length or saturation, FA uptake was instead primarily dependent on environmental concentration (Fig. 3*E*). Together, these findings indicate lipid scavenging of FAs and LPCs occurs through a class-independent, mass-action transport mechanism, which is unaffected by the FA structure.

Lipids bound to albumin are in dynamic binding equilibrium, and unbound free FAs are considered the primary form scavenged by cells (30). Thus, the availability of lipids for uptake is suggested to be influenced not only by the concentration of lipids in the environment but also by the concentration of albumin, which competes for FA binding with the cell (31). We therefore examined how BSA concentration affects FA consumption by culturing cells in media containing a fixed concentration of FA with varying amounts of BSA in the media (Fig. 3*F*). Consistent with previous reports, the consumption of FA from the media scaled proportionally with the FA:BSA ratio, where a higher FA:BSA ratio was associated with increased FA consumption, despite lower BSA available to cells (30). These data are consistent with a model in which, as the FA binding sites on BSA approach saturation, the concentration of free FA increases, leading to proportionally higher lipid consumption. Thus, higher FA:BSA ratios drive increased lipid consumption, supporting the finding that available FA concentration primarily governs consumption rates. Altogether, these data provide insight into the factors governing lipid consumption by proliferating cells and demonstrate the utility of a defined serum substitute media to isolate the variables governing nutrient uptake.

### Lipid scavenging is required for cancer cell proliferation

**Figure 4:**
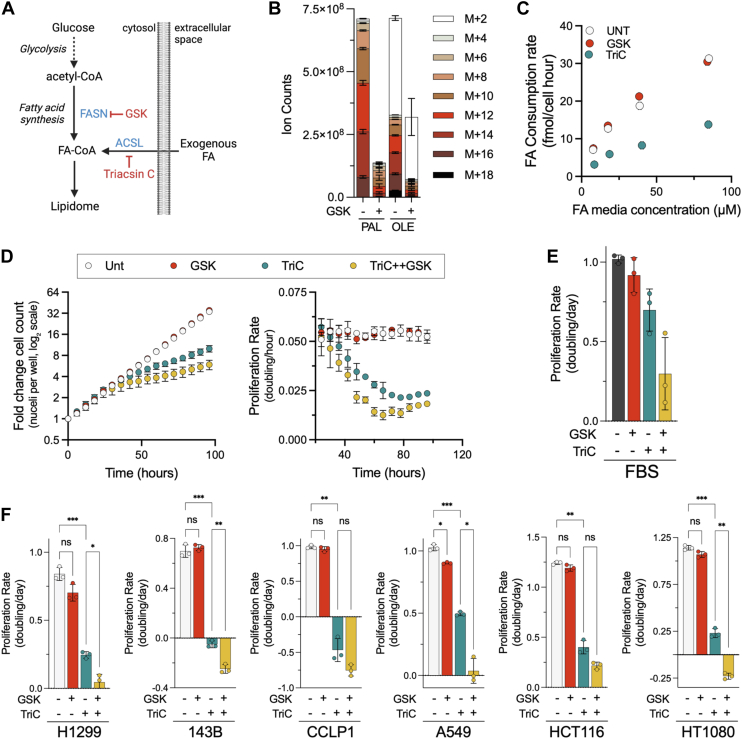
**Disruption of fatty acid (FA) activation limits lipid scavenging and impairs cancer cell proliferation.***A*, schematic of FA sources contributing to the cellular lipidome with steps inhibited by GSK2194069 (GSK) and triacsin C (TriC). *B*, isotopolog distribution of palmitate (C16:0) and oleate (C18:1) in cells cultured in U-^13^C-labeled glucose with or without GSK (200 nM) for 24 h. *C*, FA consumption rates in cells cultured in serum-free media across an FA concentration gradient, treated with either vehicle (UNT), GSK, or TriC (4 μM). *D*, fold change in cell count (*left*) and moving average of proliferation rate (*right*) for H1299 NucRFP cells grown in FA-containing serum-free media treated with GSK, TriC, or both. Fold change in cell counts (*left*) and moving average of proliferation rate (*right*). *E*, proliferation of H1299 NucRFP cells grown in 10% FBS-containing media treated with GSK, TriC (1 μM), or both, calculated using initial and final cell counts. *F*, average proliferation rate of H1299, A549, CCLP1, HCT116, 143B, and HT1080 cells grown in FA containing serum-free media treated with GSK or TriC, calculated using initial and final cell counts. Error bars represent mean ± SD (n = 3). Statistical significance was assessed using a Brown–Forsythe and Welch ANOVA tests (*F*). ns = not significant, ∗*p* < 0.05, ∗∗*p* < 0.01, and ∗∗∗*p* < 0.001. FBS, fetal bovine serum; OLE, oleate; PAL, palmitate.

Metabolic demand is typically a major determinant of nutrient consumption; however, our data indicate that lipid consumption is driven by environmental concentration (32). Thus, we next sought to explore whether perturbations to cellular metabolism in FA synthesis or FA activation influenced lipid consumption. We used GSK2194069 (GSK), a fatty acid synthase (FASN) inhibitor, or triacsin C (TriC), a pan inhibitor of the acyl-CoA long-chain (ACSL) family of proteins, which catalyze the condensation of free FAs with coenzyme A and enable their entry into metabolic pathways (Fig. 4*A*) (33, 34). Using isotope tracing, we confirmed that GSK was effective in preventing *de novo* palmitate synthesis, as evidenced by the loss of isotope incorporation from U-^13^C glucose into palmitate (16:0), the first product of FASN-mediated FA synthesis (Fig. 4*B*). GSK treatment also blocked higher order labeling species of the downstream FA metabolite, oleate (18:1), but did not abolish the amount of oleate with the M+2 isotopolog (Fig. 4*B*). These data demonstrate on-target inhibition of FASN, as cells maintained the capability to elongate/desaturate unlabeled upstream scavenged FAs to produce oleate.

We previously showed the importance of scavenged lipids in sustaining cell proliferation, prompting us to investigate how disruption to lipid metabolism influences FA sourcing. We cultured cells across a range of FA concentrations in defined media in the presence of GSK or TriC and quantified FA consumption. Inhibiting FASN-dependent lipid synthesis had no measurable impact on FA consumption, consistent with our earlier observation that lipid uptake is primarily determined by extracellular availability (Fig. 4*C*). In contrast, TriC treatment resulted in a substantial loss of FA consumption at all media concentrations, consistent with an essential role of ACSL-dependent FA activation for entry of scavenged free FAs into metabolic networks. These data indicate that FA consumption is independent from perturbations to endogenous FA synthesis and is instead reliant on intact scavenging pathways that are responsible for the uptake, activation, and trafficking of exogenous lipids.

We next explored the functional consequences of lipid scavenging and synthesis inhibition on cell proliferation with GSK and TriC treatment in defined media. GSK alone had a negligible effect on H1299 NucRFP proliferation, whereas TriC treatment modestly impaired growth (Fig. 4*D*). However, when lipid uptake capacity was constrained by TriC treatment, GSK cotreatment impaired cell proliferation, suggesting that lipid synthesis can modestly contribute to proliferation when scavenging is limited. To assess whether the relationship between scavenging and synthesis is conserved in more complex environments, we repeated these treatments in FBS-containing media (Fig. 4*E*). As in defined media, GSK had minimal effects, whereas TriC treatment alone reduced proliferation, and this effect was further enhanced by cotreatment with GSK. These findings demonstrate that the interaction between lipid synthesis and scavenging is preserved in defined and FBS-containing culture systems. Moreover, this supports the utility of defined media for metabolic studies in controlled settings, as it can recapitulate key metabolic and proliferative dependencies exhibited by cells in more complex media.

To validate the specificity of this effect, we generated H1299_NucRFP FASN KO cell lines using CRISPR–Cas9 and evaluated the proliferation effects of culturing them in different media conditions (Fig. S5*A*). Mirroring their parental cells, FASN KO cells were capable of rapid proliferation in media with 10% FBS or in serum-free media, corroborating that FASN activity is not essential when exogenous lipids are available for scavenging (Fig. S5*B*). As expected, TriC treatment moderately impaired growth of H1299 FASN KO cells, although these cells did not exhibit substantial further toxicity upon GSK cotreatment, supporting the conclusion that the proliferative defects observed from GSK in TriC-treated parental cells result from its impairment of FASN (Fig. S5*B*). Together, these data indicate that, while FA synthesis capacity appears unable to meet lipid synthesis demands on its own, it can modestly contribute to proliferation when lipid uptake is constrained.

Finally, to assess whether the relationship between lipid synthesis and scavenging is conserved across various cell lines, we tested the effects on cell proliferation rate of 143B, CCLP1, A549, HCT116, and the fibrosarcoma cancer cell line HT1080 grown in the serum-free media when treated with GSK, TriC, or both (Fig. 4*F*). As with H1299 cells, all cell lines were insensitive to GSK alone, exhibited moderate sensitivity to TriC, and often showed synergistic proliferative impairment when cotreated with both inhibitors (Fig. 4, *D*–*F*). This conserved phenotype across various cancer cell lines highlights a shared metabolic dependency in which exogenous lipids are critical to meet the metabolic demands for proliferation, and *de novo* lipid synthesis becomes essential only when scavenging is impaired.

## Discussion

FBS, a ubiquitous additive in culture media, supports proliferation across numerous cell types; however, the specific serum components needed for growth are not well defined. Here, we combined live-cell imaging with LC–MS to show that metals and albumin-associated lipids in serum are crucial factors for sustaining cancer cell proliferation in culture. We observed that albumin-bound lipids, such as FA, LPC, and LPE, were rapidly consumed from the media, whereas lipoprotein-transported lipids were minimally depleted during proliferation. Furthermore, we found that a defined mixture of insulin, transferrin, selenium, ethanolamine, and various trace metals was an effective serum replacement (predominantly for providing metals) when combined with albumin-associated lipids. This system therefore provides a tool to investigate lipid consumption in a chemically defined and controllable environment. Using these defined media, our results support a model whereby albumin-associated lipid consumption follows mass-action kinetics that are independent of FA structure and exhibit minimal competition between lipid classes. Notably, this uptake did not correspond to lipid demand, as inhibiting *de novo* lipid synthesis had minimal effects on lipid scavenging. However, direct disruption of components in the scavenging pathway blocked lipid uptake, impaired cell proliferation, and sensitized cells to disruption of synthesis inhibition. Together, our findings demonstrate that lipid scavenging, not *de novo* synthesis, is the dominant source of lipids during cancer cell proliferation in nutrient-replete environments.

Our results contribute to the development of serum-free tissue culture media, which could have benefits for research beyond the cost savings and ethical considerations of omitting FBS (35). For instance, recent data have shown that many genetic dependencies are influenced by the nutrient composition in the microenvironment; therefore, serum-free conditions provide an opportunity to uncouple the numerous metabolic variables inextricably supplied by FBS (10, 36). Although it could be argued that the molecular complexity of FBS more closely models *in vivo* conditions, the composition and abundance of bioactive molecules are poorly defined and may not accurately reflect the extracellular microenvironment of adult human tissues. This could obscure phenotypes relevant to tumor biology or metabolic vulnerabilities. In agreement with other studies, we also find that FBS lots exhibit substantial variation in their metabolomic profiles, particularly lipids, which also significantly differ from human serum (5, 21). Our defined formulation offers a more defined and reproducible alternative to serum and may facilitate future studies using genetic or pharmacologic screens in cells grown in defined media to uncover which metabolic and signaling pathways are buffered by serum constituents. Thus, this approach may identify additional serum factors that support proliferation in context-specific ways.

A major finding from using a serum-free media to explore lipid consumption dynamics is that FA scavenging is primarily driven by the concentration of exogenous lipids rather than by selective uptake based upon lipid structure in proliferating cells. Indeed, treatment of cells with increased levels of FAs is well described to cause meaningful changes to cell physiology, including driving lipid droplet formation, causing lipotoxicity when excess saturated FAs accumulate intracellularly, and promoting ferroptosis when excess polyunsaturated FAs are enriched in membrane phospholipids (37, 38, 39, 40). These vulnerabilities, which are governed by the extracellular lipid composition, support our conclusion that FA consumption rate is primarily determined by exogenous abundance rather than metabolic need. Although not directly related to these lipotoxic outcomes, our findings raise the possibility that lipid composition in the tumor microenvironment could modulate lipid stress sensitivities. Together, these data highlight the potential utility of nutritional approaches that may modify the tumor lipid microenvironment to promote cancer cell lipid states that may be favorable for therapeutic targeting.

The selective consumption of albumin-associated lipids identified in this study may reflect differences in lipid bioavailability and accessibility, rather than lipid class–specific uptake. Lipids transported in lipoprotein complexes are sequestered in the core or in the lipoprotein membrane and less freely interact with the cell. In contrast, albumin-associated lipids exist in dynamic binding equilibrium with albumin, allowing a subset to exist as free lipids in the solvent that are available for uptake *via* passive diffusion or through interaction with lipid transporters on the cell surface. While proteins involved in FA and LPC transport (*e.g.*, CD36 and MFSD2a) have been implicated to exhibit substrate preference based upon FA structure, it is unclear if this leads to relevant differences in substrate consumption during cell proliferation (41, 42). Consistent with the minimal selectivity of scavenging based on fatty acyl structure, screens aimed at identifying lipotoxicity modifiers have predominantly uncovered factors that regulate intracellular lipid homeostasis, such as those involved in lipid droplet formation and phospholipid remodeling, rather than lipid transporters (43, 44). Moreover, because lipid consumption is proportional to exogenous concentrations, interpretations of selective lipid uptake may reflect differences in initial abundance and lipid stability.

Prior studies have shown that the relative contribution of lipid scavenging and *de novo* synthesis varies by cancer type and oncogenic context (45). However, despite the increase in lipogenic enzyme expression commonly observed in cancer cells, our data underscore a critical role of exogenous lipids in fulfilling the lipid requirement for proliferation in the context of growth in culture (46, 47). Indeed, exogenous lipid sources sustained serum-free cultivation of cells, and our results are consistent with the minimal effects of disruption to *de novo* lipid synthesis on cell proliferation when exogenous lipids are available (48). The critical nature of lipid scavenging is further supported by the modest clinical efficacy of FASN inhibitors as a monotherapy for cancer treatment, perhaps because lipid-rich microenvironments may enable efficient lipid scavenging that diminishes the importance of lipid synthesis *in vivo* (49, 50, 51). Indeed, cells cultured in physiologically relevant concentrations of lipids, which is approximately an order of magnitude greater than what is present in media containing 10% FBS, have been shown to preferentially incorporate scavenged lipids into cellular membranes during proliferation (21, 25). We further identify the ACSL family of proteins as critical mediators of exogenous FA scavenging through metabolic trapping, as disruption of FA activation with TriC impairs FA scavenging. This is consistent with the essentiality of FA condensation with coenzyme A for lipid entry into the cellular metabolic pathways (52). Prior studies using genetic and pharmacological perturbations to ACSL family members indicate these proteins are crucial for FA incorporation into neutral lipids and phospholipids, and our results extend this role to directly implicate this family of proteins in the FA scavenging pathway (53). Interestingly, only when FA scavenging was constrained could FA synthesis detectably contribute to cell proliferation, and this response varied across cell lines. Together, these findings suggest that targeting lipid scavenging could be a more effective therapeutic approach to target the increased lipid demand of proliferating cancer cells, though any such strategy would have to overcome the redundancy of alternate albumin-bound lipid sources.

While our serum-free media enable improved experimental control over the environmental variables that influence proliferation, all experiments were conducted in cell culture systems that do not model the complexity of the cellular environment *in vivo*. For example, relevant factors that influence cell growth *in vivo*, such as stromal, vascular, and immunologic interactions, can shift the metabolic dependencies for proliferation and are not modeled in this cell culture system (54, 55, 56). Thus, the lipid requirements identified here will need *in vivo* validation. Moreover, while our system identifies the minimal components sufficient to support cancer cell proliferation *in vitro*, other FBS-derived molecules—such as growth factors, cytokines, or additional metabolites—may become essential under different physiological or stress conditions, including immune evasion or nutrient limitation (57). Nonetheless, this defined media system provides both a reproducible serum replacement and a tractable platform for dissecting mechanistic metabolic dependencies under defined conditions.

## Experimental procedures

### Reagents

Unlabeled oleic acid (O1008) and palmitic acid (P0500) used in proliferation experiments were purchased from Sigma. U-13C oleic acid (CLM-460), U-13C palmitate (CLM-409-0.1), and d11-arachidonic acid (10006758) isotope standards were obtained from Cambridge Isotopes. 5-13C oleic acid (9004089), 4-13C palmitate (30550), LPC 18:1 (20959), and LPC 16:0 (10172) used in consumption assays were purchased from Cayman Chemical. LPE 18:1 (846725P), LPE 16:0 (856705P), LPC 18:1-d7 (791643), LPE 18:1-d7 (791644), and lipids used to generate calibration curves SPLASH LIPIDOMIX Mass Spec Standard (330707), LightSPLASH LIPIDOMIX Quantitative Mass Spectrometry (MS) Primary Standard (330732) were purchased from Avanti Polar Lipids. The chemical inhibitors used in this study, GSK (SML1259) and TriC (2472), were purchased from Sigma and Tocris, respectively. FBS lots used for serum lipid comparison were purchased from Cytiva (SH30396.03; Lot no.: AH30469640 and AK30775909), Gibco (A52567-01; Lot no.: M3009140RP), and Corning (35-077-CV; Lot no.: 22023001).

### Cell culture

Cell lines were acquired from the American Type Culture Collection (143B, H1299, HCT116, HT1080, A549, and Jurkat), JCRB Cell Bank (OCUG1), or as a gift from laboratories at the Fred Hutch, including Dr Supriya Saha, Fred Hutch (CCLP1, SSP25), Dr Stephen Tapscott (C2C12), Dr Alice Berger (AALE), and Dr Adam Geballe (HFF1). H1299 NucRFP cells were previously generated, as described (58). Cell identities were confirmed by satellite tandem repeat profiling, and cells were tested and found to be free from mycoplasma (MycoProbe; R&D Systems). Cells were maintained in DMEM (Gibco; 50-003-PB) supplemented with 3.7 g/l sodium bicarbonate (Sigma–Aldrich; S6297), 10% FBS (Cytiva; SH30396.03), and 1% penicillin–streptomycin solution (Sigma; P4333). Cells were incubated in a humidified incubator at 37 °C with 5% CO_2_.

### Reagent preparations

Numerous cell culture additives containing ITS are currently available, often containing supplements beyond its namesake additives, resulting in heterogeneous ITS formulation offerings that are sometimes referred to as “ITS-plus.” The ITS mixture used here was that of ITS Solution Plus (100×), Animal Free (GenDEPOT; CA202), which was used as purchased or made in-house by assembling its components (or subsets thereof, as described) into a 100× stock solution containing 0.5 g/l recombinant insulin (Sigma; I9278), 0.6 g/l recombinant transferrin (Sigma; T8158), 0.67 mg/l of sodium selenite (Sigma; 214485), 10 mg/l ethanolamine (Sigma; E0135), 2.04 mg/l ferric citrate (Sigma; F3388), 863 μg/l of zinc sulfate (Sigma; 83265), and 1.6 μg/l of copper(II) sulfate (Sigma; C8027) in water. These solutions were used as a spike-in supplement at 1% into culture media. Lipid mixes were made by dissolving lyophilized lipids in 100% MeOH to a final concentration of 100 mM, and lipid solutions were aliquoted and stored in glass vials at −20 °C in glass. Lipid solutions were then diluted to a final concentration of 200 μM in DMEM containing 50 μM BSA fraction V, heat shock, FA free (Sigma; 3117057001) and placed in a flask and shaken at 37 °C for 1 h. Lipid solutions (200 μM) were then diluted in DMEM to the final working concentration. Unless otherwise noted, lipid mixes were combined at a 95:5 M ratio of oleate:palmitate FA chains for both FA mix and LPC mix, whereas LPE lipid mix was a 60:40 M ratio of oleate:palmitate FA chains.

### IncuCyte/proliferation experiments

H1299 NucRFP cells were trypsinized (Corning; 25-051-CI), resuspended in media, counted (Beckman Coulter Counter Multisizer 4 or Nexcelom Auto T4 Cellometer), and seeded overnight onto 24-well dishes (Thermo; 142475) with an initial seeding density of 5000 cells/well. After overnight incubation, wells were washed three times in PBS, and 1 ml of treatment media was added. Experiments were conducted in DMEM (Corning; 50-013-PB) without pyruvate, supplemented with 3.7 g/l sodium bicarbonate (Sigma; S5761) and 1% penicillin–streptomycin solution (Fisher; 15-140-163) and 1 mM sodium pyruvate (Sigma–Aldrich; P8574) and supplemented with the indicated treatments. Plates were imaged in real time using the IncuCyte S3, at 20× magnification with a 400 ms exposure for the red channel, and set to capture images every 6 h. Fold change was calculated as the nuclei count for each time point relative to the initial nuclei count in the corresponding well. Proliferation rates were calculated using the equation: Proliferation rate = log_2_ (final cell count/initial cell count)/time elapsed. The IncuCyte moving averages represent the mean proliferation rate calculated between consecutive time points, averaged over 24-h intervals. For standard proliferation assays, initial and final cell counts were collected and used to calculate proliferation rates.

### Consumption experiments

Cells were seeded on 6-well dishes (Corning; 087721B) in DMEM containing 10% FBS. The following day, the wells were washed three times in PBS before swapping cells into 2 ml of the indicated media. Plates were placed back in the incubator for 1 h to allow for the cells and media to equilibrate. For consumption experiments in 10% FBS–DMEM, media were collected at the indicated times and cells were counted. For consumption experiments in the serum replacement media, cell counts were collected from a parallel plate at time 0, and 100 μl of media was collected at 2-h intervals for 6 h. At the final time point, cell counts were collected from the well to calculate cell hours and proliferation rates during the assay. Media concentrations were plotted on the *y*-axis over time. The *x*-axis represented cumulative cell hours, calculated based on cell proliferation dynamics using the following formula:

$$Cell\;hours=\left(\frac{Starting\;cell\;count}{Proliferation\;rate*\ln\left(2\right)}\right)*\left(2^{Proliferation\;rate*time}-1\right)$$

This formula accounts for exponential cell growth during consumption assays to estimate the area under the curve of cumulative proliferating cells over time. To calculate consumption rate, cell hours were plotted on the *x*-axis and the moles of each lipid were plotted on the *y*-axis. A linear regression analysis was performed in GraphPad Prism (GraphPad Software, Inc), and the slope of the fitted line was used to determine the lipid consumption rate, expressed as the rate of lipid consumed per cell hour, with the slope’s standard error representing measurement uncertainty.

### Lipidomic sample preparations

Media samples were extracted using a single-phase extraction protocol, where 30 μl of media was combined with 270 μl of 2:1 ratio of ethyl acetate (Thermo; 022912.K2):2-propanol (Sigma; 34863) containing isotopically labeled lipid standards in glass conical vials (MicroSolv Technology; 9512S-0CV-T-RSD), resulting in a final extraction solvent of 6:3:1 ethyl acetate:2-propanol:water. Samples were vortexed for 10 min and centrifuged at maximum speed to pellet debris. The supernatant (200 μl) was transferred to a clean glass vial before drying down using a refrigerated vacuum concentrator (Fisher; 10269602). Once dry, lipids were resuspended in 100 μl of 65:30:5 acetonitrile (Sigma; 34851):2-propanol:water and transferred to glass autosampler (Fisher; 03-452-330) vials for MS analysis. MS runs were analyzed using Skyline software for metabolomics analysis (59). Semitargeted lipidomics analysis of serum lipids was normalized to Avanti SPLASH deuterated standard for each class, whereas targeted consumption assays included standards for each individual lipid.

### Isotope tracing

H1299 cells were seeded in a 6-well dish at an initial density of 2 × 10^5^ cells per well. The following day, cells were washed twice with PBS and swapped to DMEM without glucose, glutamine, pyruvate, or phenol red (Sigma; D5030) supplemented with 10% dialyzed FBS (Sigma; F0392), 1% penicillin–streptomycin, 25 mM U-^13^C glucose (Cambridge Isotopes Laboratory; CLM-1396), and 4 mM ^12^C glutamine (Sigma; G5792). GSK-treated cells were supplemented with 200 nM of GSK, and all plates were placed back in an incubator for 24 h before extraction.

### Complex lipid analysis

A 2 μl injection of sample (held at 10 °C in the autosampler) was made onto a hypersil gold column (1.0 × 150 mm with a 1 × 10 mm guard, at 50 °C) and was eluted (from 32% to 97% “B” over a 36-min total run time) using a multistep gradient. The composition of the mobile phases used consists of “A” = water:acetonitrile at 60:40 v/v with formic acid at 0.1% and 10 mM ammonium formate and “B” = acetonitrile:isopropanol at 10:90 v/v containing 10 mM ammonium formate with formic acid at 0.1%. Eluted analytes were analyzed with a Q-Exactive HF-X at 120 k resolution in the MS1 mode in both positive and negative polarities in the same run, with ddMS2 performed on the top 15 precursors at 30 k resolution with stepped normalized collision energies of 25 and 30, respectively. Identification of lipids was made by accurate mass comparison to an in-house database of standards and validated by comparison of the MS2 spectra to predicted fragmentation patterns. Prior to data collection, the performance of the instrument and chromatographic system was evaluated for retention time consistency and signal intensity using an injection of 1,2-distearoyl-*sn*-glycero-3-phosphocholine (PC 18:0) solubilized in 50:50 (v/v) mobile phase A and B. In addition, instrument performance and mass calibration are evaluated throughout the sample run by a daily injection of LIPIDOMIX SPLASH mix (Avanti Polar Lipids) containing 14 deuterium-labeled lipid species.

### FA analysis

Post extraction, FA samples were resuspended in acetonitrile/isopropanol/water 65:30:5 v/v/v, and a 2 μl injection was made onto a Kinetex C8 core-shell column (2.1 × 100 mm, 2.6 μm, and attached 2.1 mm i.d. SecurityGuard Ultra C8 guard column). The autosampler is held at 10 °C, whereas the column is kept at 40 °C through the run. The mobile phase composition consisted of “A” = water with acetic acid (at 0.1%) mixed with 5% (by volume) of mobile phase “B,” where “B” = acetonitrile/methanol/0.1% acetic acid (80:15:5 v/v/v). A semi-isocratic gradient is used to elute the analytes off the column, beginning at 20% for 0 to 1 min, followed by a rapid increase of “B” to 66%, which is then held for 6.5 min, followed by a further increase of “B” to 100%. Finally, a short wash phase is followed by a re-equilibration phase of 7 min at starting conditions (20%), with total injection-to-injection times of 22 min. Eluted analytes were detected with the Q-Exactive HF-X in MS1 mode over a mass range of 210 to 600 *m/z* using negative polarity. Analytes were identified by accurate mass comparison to an in-house database of standards. Prior to analysis and throughout the sample series, instrument performance is evaluated using a mixture (diluted 1:4 using 50:50 mobile phase “A” and “B”) containing 10 monounsaturated FAs at 10 ng/μl each (Cayman Chemical). Mass calibration, retention times, and peak intensities were monitored to ensure consistent performance.

### Generation of KO cell lines

The protocol was adapted from a previously described protocol (60). Three chemically synthesized 2′-O-methyl 3′phosphorothioate–modified single guide RNA (sgRNA) sequences targeting the gene of interest were purchased (Synthego) and are provided below. Each sgRNA was resuspended in nuclease-free water, combined with SF buffer (Lonza; V4XC-2032), and sNLS-spCas9 (Aldevron; 9212). H1299 NucRFP cells (2 × 10^5^) were resuspended in the resulting solution containing ribonucleoprotein complexes and electroporated using a 4D-Nucleofector (Amaxa; Lonza) program EW-127. Nucleofected cells were then moved to a 12-well plate (Corning; 3513) and, after achieving confluence, were single-cell cloned by limiting dilution by plating 0.5 cells/well in a 96-well plate. Gene KO was confirmed using Western blots on the nucleofected pool, and single-cell clones were generated by limiting dilution and validated by Western blot prior to use in this study.

#### FASN sgRNA sequences:

(1): CAUCUCCAAGACCUUCUGCC

(2): GCCCCCUUCAGCACUGCCUC

(3): UGCUUGGACGCAGGUGCUUG

### Western blotting

Protein lysates were harvested in radioimmunoprecipitation assay buffer (Sigma; R0278) supplemented with protease inhibitors (Fisher; A32953). Protein concentration was determined using a bicinchoninic acid assay (Fisher; 23225) using BSA as a protein standard. Equal amounts of protein were denatured with Bolt 4x Loading Dye (ThermoFisher; B0007) and Bolt 10× reducing agent (ThermoFisher; B0004), heated at 95 °C for 5 min, and loaded onto a 3% to 8% NuPAGE Tris–acetate gel (ThermoFisher; EA0375BOX). After electrophoretic separation, proteins were transferred onto a 0.22 mm nitrocellulose using iBlot2 transfer stacks (Fisher; IB23001) and transferred at 25 V for 10 min. Membranes were blocked with 5% milk in Tris-buffered saline with 0.1% Tween-20 (TBS-T) and incubated at 4 °C overnight with the following antibodies: anti-FASN (Cell Signaling; 3180S; 1:1000 dilution), anti-Vinculin (Sigma; SAB4200729; 1:10,000 dilution). The next morning, membranes were washed three times with TBS-T, and the following secondary antibodies were added: 800CW Goat anti-Mouse IgG (LiCOR; 926-32210; 1:15,000 dilution) and 680RD Goat anti-Rabbit IgG (LiCOR; 926-68071; 1:15,000 dilution). Membranes were washed three more times with TBS-T and imaged on a LiCOR Odyssey Near-Infrared imaging system. FASN antibody specificity was validated by band molecular weight and loss of immunoreactivity following genetic disruption to the antigen.

### Quantification and statistical analysis

All graphs and statistical analyses were performed in GraphPad Prism 9.0. Technical replicates, defined as parallel biological samples independently treated, collected, and analyzed during the same experiment, are shown. Experiments were verified with independent repetitions showing qualitatively similar results. Where applicable, replicate data are shown as individual points, and all error bars represent SD unless otherwise noted. Details pertaining to all statistical tests can be found in the figure legends.

## Data availability

Raw and processed data are provided as supporting information files. Raw lipidomics data will be made publicly available through the Metabolomics Workbench repository (accession no.: 6272).

## Supporting information

This article contains supporting information.

## Conflict of interest

The authors declare that they have no conflicts of interest with the contents of this article.

## Supporting information

Supporting Figures

Table S1 Raw data for live cell imaging and cell count experiments.

Table S2 Processed data for lipidomics experiments.
