# Supplementary material for: Defined media reveal the essential role of lipid scavenging in supporting cancer cell proliferation
Source: J Biol Chem. 2025 Sep 8;301(10):110693. doi: 10.1016/j.jbc.2025.110693 (PMC12547238; doi:10.1016/j.jbc.2025.110693)
Supplement: Supporting Figures [file mmc1.pdf]

Supporting Information

Title: Defined media reveals the essential role of lipid scavenging in supporting cancer cell proliferation

Authors: Oliver J. Newsom[1], Eric Zheng[1], Lucas B. Sullivan[1*]

Affiliations: [1]Human Biology Division, Fred Hutchinson Cancer Center, Seattle, WA, 98109, USA.
*Correspondence to: lucas@fredhutch.org.

Material included:
Supplementary Figures 1-5

Supplementary Tables 1 & 2 provided separately

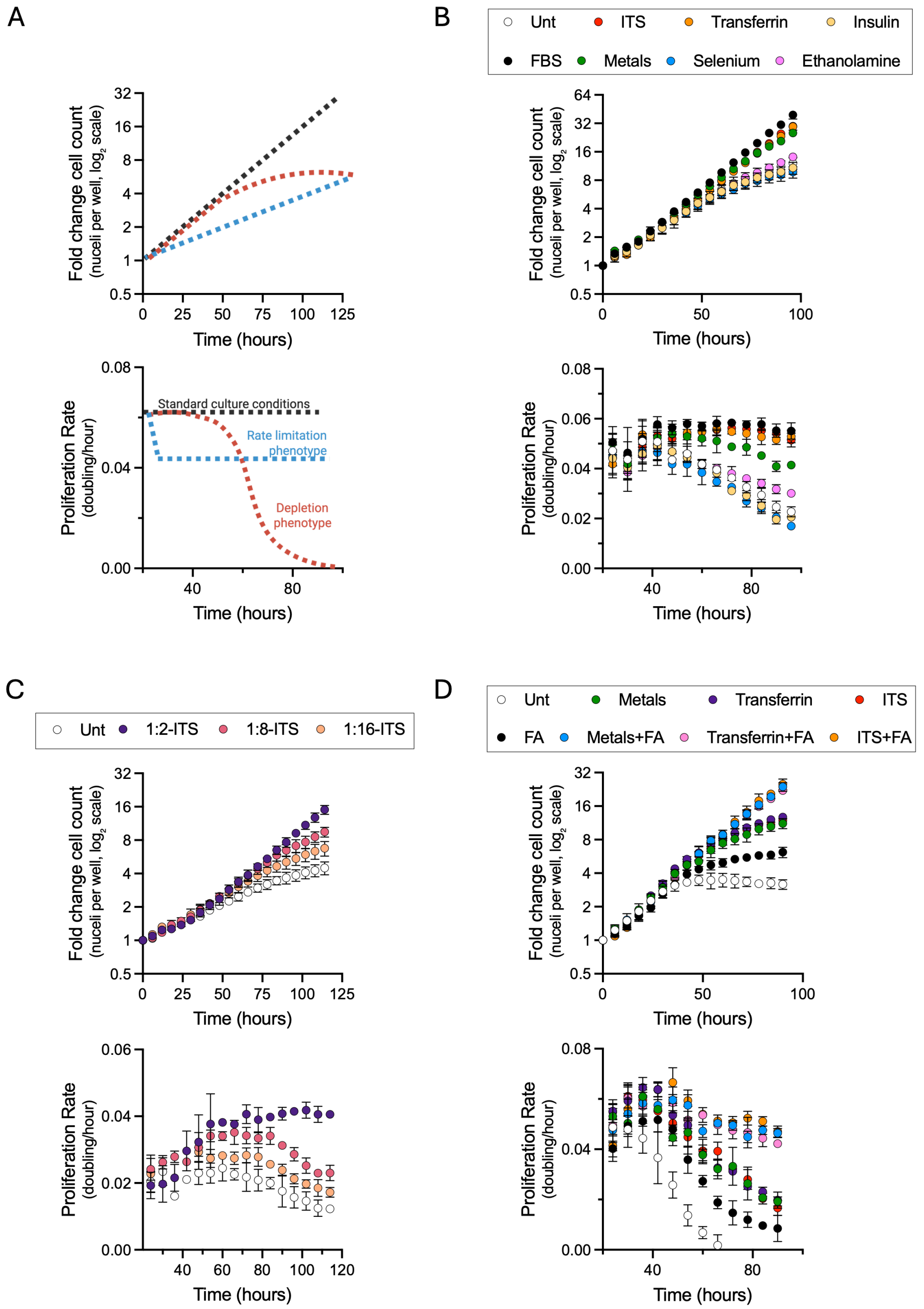

Supplementary Figure 1. Serum-restriction results in a depletion phenotype.

(A) Predicted changes in cell growth kinetics under two theoretical modes of proliferation inhibition. Fold change in cell counts (top) and moving average of proliferation rate (bottom). The blue dotted line reflects a rate limitation phenotype (consistently slowed), while the red dotted line which reflects a depletion phenotype (progressive loss of proliferation over time). These patterns can appear identical when only counting initial and final timepoints are measured. **(B)** Growth kinetics of cells cultured in 0.5% FBS supplemented with individual components of the ITS mix. Fold change in cell counts (top) and moving average of proliferation rate (bottom). **(C)** Growth kinetics of cells cultured in 0.5% FBS supplemented with decreasing concentrations of the complete ITS mix. Fold change in cell counts (top) and moving average of proliferation rate (bottom). **(D)** Growth kinetics of cells cultured in DMEM alone, ITS, transferrin, or trace metals (zinc, iron, and copper) supplemented with or without FA. Fold change in cell counts (top) and moving average of proliferation rate (bottom). Error bars represent mean ± SD (n = 3).

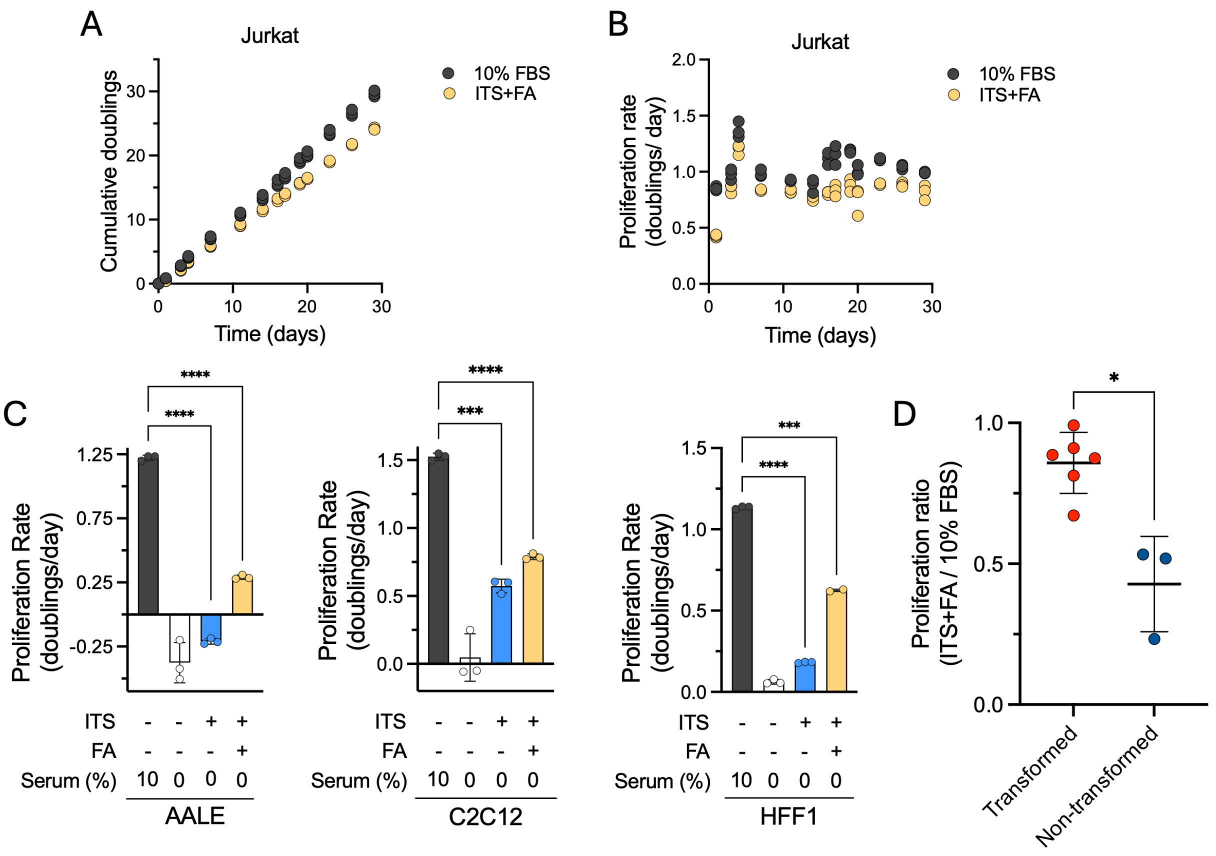

Supplementary Figure 2. Serum-free media supports transformed but not non-transformed cell growth.

(A-B) Jurkat cell proliferation in ITS+FA compared to FBS-containing media, assessed by cumulative population doublings over time (A) and proliferation rate (B). **(C)** Proliferation rates of AALE, C2C12, and HFF1 cells cultured in DMEM alone, 10% FBS, ITS, or ITS and FA mix (ITS+FA), calculated using initial and final cell counts**. (D)** Comparison of proliferation rates in transformed (H1299, 143B, CCLP1, A549, HCT116, and Jurkat) verses and non-transformed (C2C12, AALE, HFF1) cells cultured in ITS+FA compared to FBS-containing media. Error bars indicate mean ± SD (n=3, except for (D) in which the transformed cell group had an n=6). Abbreviations: ITS, insulin–transferrin–selenium mix that also contains ethanolamine and trace metals; FA, fatty acid mix. Statistical significance was assessed using an Brown-Forsythe and Welch ANOVA tests (C) and Welch t-test (D). ns = not significant, *p < 0.05, ****p < 0.0001.

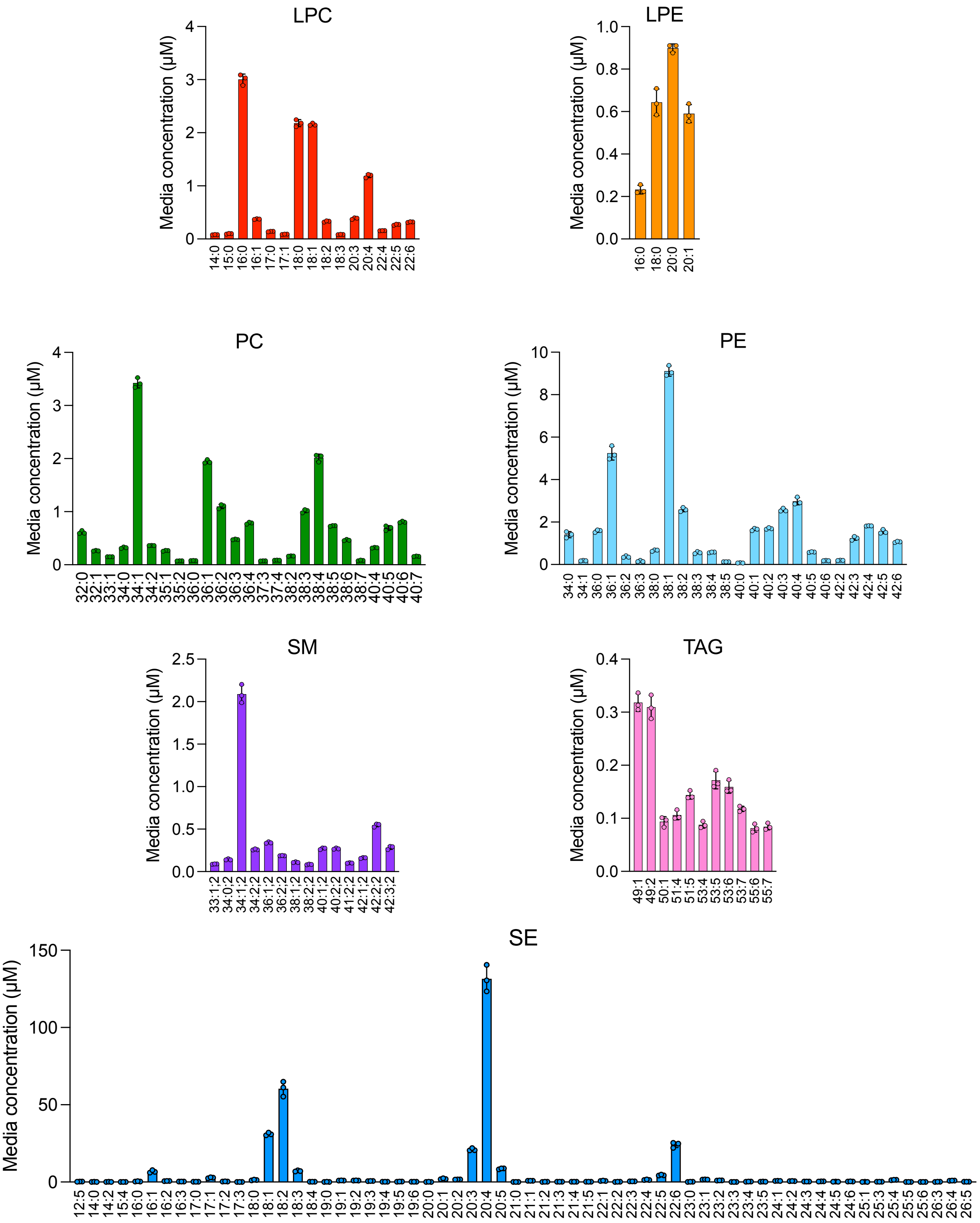

Supplementary Figure 3. Lipid profiles of FBS.

Lipid species that constitute the top 99% of the total lipid pool detected in 10% FBS containing media. The x-axis indicates the number of carbons and the number of double bonds present in each lipid species. Error bars represent mean ± SD (n = 3). Abbreviations: SE, sterol esters; PE, phosphatidylethanolamine; PC, phosphatidylcholine; SM, sphingolipids; TAG, triacylglycerols; LPC, lysophosphatidylcholine; LPE, lysophosphatidylethanolamine.

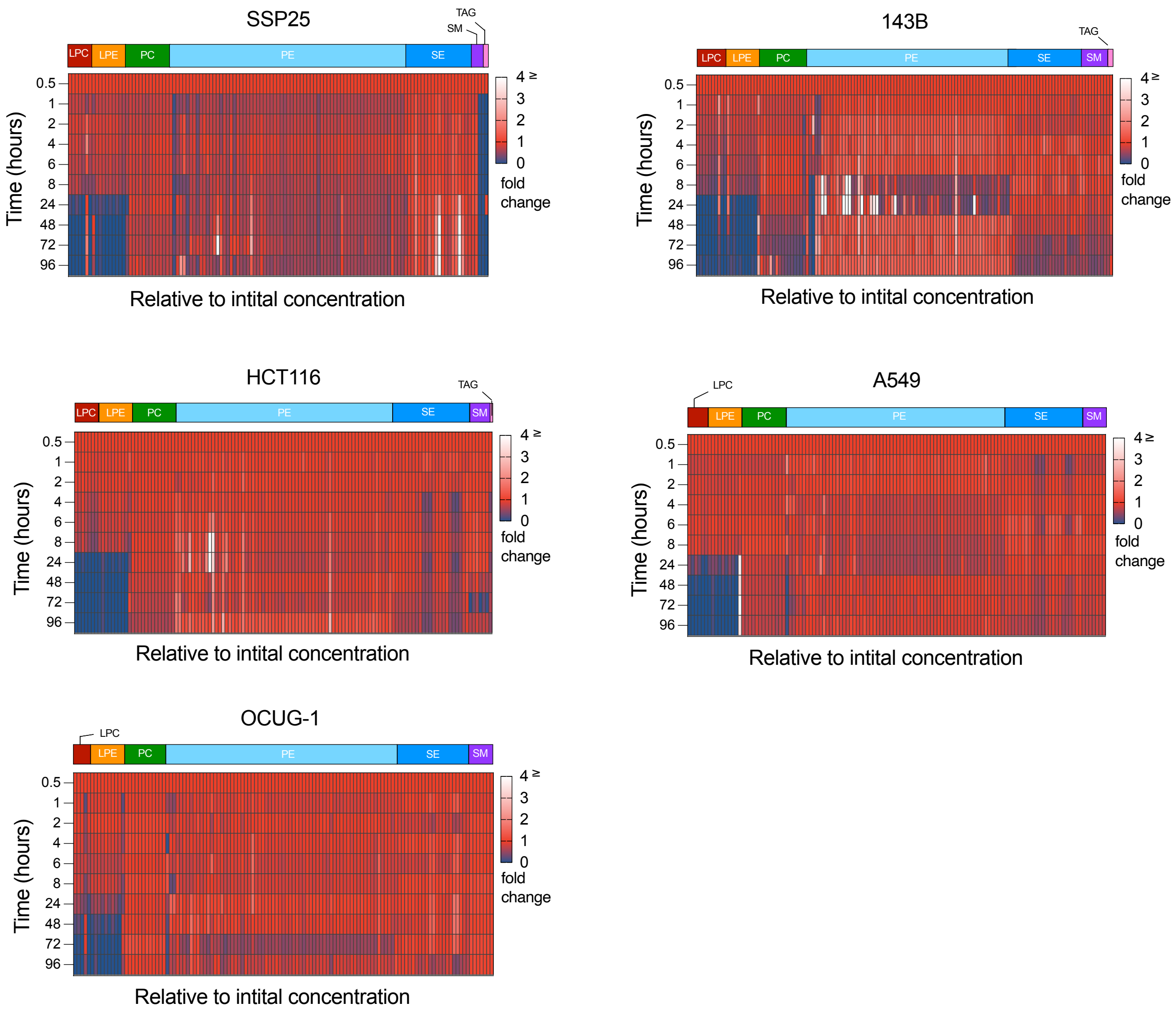

Supplementary Figure 4. Lipid consumption patterns are conserved across cell lines. Media lipid depletion from 10% FBS containing media for cholangiocarcinoma (SSP25), osteosarcoma (143B), non–small cell lung cancer (A549), colon cancer (HCT116), and gallbladder carcinoma (OCUG-1).

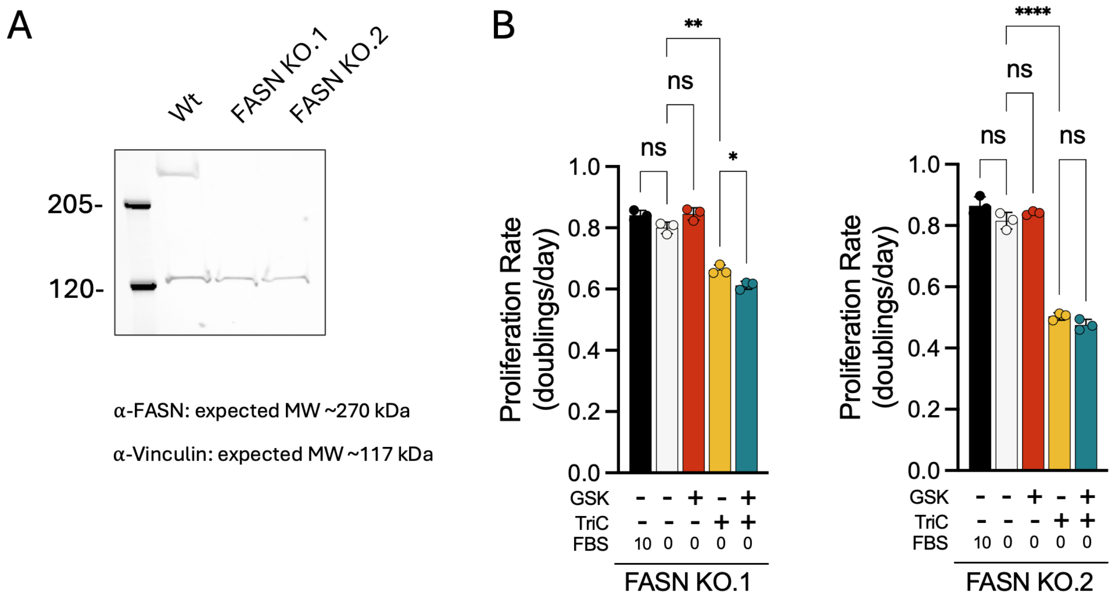

Supplementary Figure 5. Genetic deletion of FASN phenocopies pharmacologic inhibition and confirms its contribution to proliferation.
(A) Western blot confirming CRISPR-Cas9-mediated knockout of FASN in H1299 NucRFP cells. Expected molecular weight of FASN is approximately 270 kilodalton; the expected molecular weight of vinculin is 117 kilodaltons. **(B)** Proliferation of FASN knockout cells cultured in either 10% FBS or ITS+FA containing media treated with GSK (200nM), TriC (4uM), or the combination. Error bars indicate mean ± SD (n=3). Statistical significance was assessed using Brown-Forsythe and Welch ANOVA tests (B). ns = not significant, **$p < 0.05$, **$p < 0.01$, ***$p < 0.001$, ****$p < 0.0001$.
